# The Metabolic and Ecological Interactions of Oxalate-Degrading Bacteria in the Mammalian Gut

**DOI:** 10.3390/pathogens2040636

**Published:** 2013-12-06

**Authors:** Aaron W. Miller, Denise Dearing

**Affiliations:** Department of Biology, University of Utah, 257 South 1400 East, Salt Lake City, UT 84112, USA; E-Mail: dearing@science.utah.edu

**Keywords:** oxalate-degrading bacteria, gut microbiota, plant secondary compounds, oxalate, biotransformation

## Abstract

Oxalate-degrading bacteria comprise a functional group of microorganisms, commonly found in the gastrointestinal tract of mammals. Oxalate is a plant secondary compound (PSC) widely produced by all major taxa of plants and as a terminal metabolite by the mammalian liver. As a toxin, oxalate can have a significant impact on the health of mammals, including humans. Mammals do not have the enzymes required to metabolize oxalate and rely on their gut microbiota for this function. Thus, significant metabolic interactions between the mammalian host and a complex gut microbiota maintain the balance of oxalate in the body. Over a dozen species of gut bacteria are now known to degrade oxalate. This review focuses on the host-microbe and microbe-microbe interactions that regulate the degradation of oxalate by the gut microbiota. We discuss the pathways of oxalate throughout the body and the mammalian gut as a series of differentiated ecosystems that facilitate oxalate degradation. We also explore the mechanisms and functions of microbial oxalate degradation along with the implications for the ecological and evolutionary interactions within the microbiota and for mammalian hosts. Throughout, we consider questions that remain, as well as recent technological advances that can be employed to answer them.

## 1. Introduction

Mammalian physiology is greatly influenced by complex, metabolic interactions between the mammalian host and the microbiota. Outnumbering the host’s own genes 150-fold, the microbiota is primarily concentrated in the gastrointestinal (GI) tract [1,2,3]. Here, it exists in both obligate and facultative symbioses with the host and shares both functions and gene products essential to “normal” mammalian physiology and metabolism [1,3,4,5,6,7]. Disruptions of the host-microbe metabolic network can occur through antibiotic use or through diet that can lead to dysbiosis and disease [3,8,9,10]. Several diseases have been correlated with altered gut microbiota, including obesity, diabetes, intestinal disorders, and metabolic syndrome [11,12,13].

The biotransformation of toxins is an important function provided by gut microbes that contributes to the normal physiology of the host. Many commonly consumed plants contain plant secondary compounds (PSCs) that can alter the physiological homeostasis of mammals [14,15,16,17,18]. These PSCs can lead to mortality or disease when consumed in high doses [14,15,16,17,18]. Several mechanisms may be employed to minimize the negative effects of toxic PSCs, such as limiting consumption or absorption, or enhancing biotransformation and excretion [19]. The immense metabolic plasticity inherent within the gut microbiota provides a strong potential for the biotransformation of dietary toxins [20,21]. To date, dozens of bacterial species have been isolated from the GI tract of mammals that are capable of biotransforming toxic PSCs into non-toxic by-products. Examples of classes of PSCs that can be metabolized by microbes include mimosine, tannins, and phenolic compounds [22,23,24,25,26]. The microbial contribution to overcoming challenges associated with consuming plants is well known and is perhaps reflected in gut microbiota diversity, which is greatest in mammalian herbivores followed by omnivores and carnivores [21,27,28].

The toxin oxalate presents an ideal system to understand the host-microbe metabolic interactions that maintain homeostatsis in the face of a toxic challenge. Oxalate entering into the body is primarily derived from dietary sources. However, several dietary precursors such as glyoxalate, glycine, or hydroxyproline can be metabolized to oxalate by the mammalian liver to produce NADH [15,29,30,31,32,33,34,35]. Consumption of oxalate or its precursors can have a considerable impact on mammals. In its dianionic form, oxalate is a strong chelating agent that readily binds to free metallic cations such as calcium [36]. Mortality from oxalate ingestion can occur with a single exposure, characterized by azotemia, hypocalcemia, hemorrhaging of visceral organs, and the presence of oxalate crystals in the kidneys [37]. However, sublethal effects including corrosion of the mouth and gastrointestinal tract, gastric hemorrhaging, diarrhea, kidney stones, and inflammation can significantly impair a mammal’s ability to function normally and thus have an indirect effect on mortality [38,39,40,41,42].

Despite the toxicity and widespread occurrence of oxalate, mammals do not produce enzymes capable of biotransforming the compound and instead rely on limiting absorption, excretion, or microbial degradation [29,33,43,44]. Microbial oxalate degradation in the GI tract reduces oxalate circulating in the blood and is a negative risk factor for kidney disease [45,46]. Thus, significant host-microbe metabolic interactions maintain the balance of oxalate in the body and disruptions in this metabolic network can lead to disease. In this review, we explore the effects of oxalate on whole microbial communities and the ecological interactions that facilitate the degradation of oxalate by the gut microbiota of mammals. First, we will define the flow of oxalate through the body and the ecological potential for oxalate degradation by the microbiota along the GI tract. Next, we review the mechanisms and functions of microbial oxalate degradation and the diversity of known oxalate-degrading bacteria within the mammalian gut. We further address the short-term population dynamics of the microbiota associated with oxalate exposure, as well as long-term evolutionary changes. Throughout the review, we will identify gaps in current literature and offer new perspectives and tools on how to address them.

## 2. The Flow of Oxalate in the Body

Knowing how oxalate moves through the body is imperative to elucidating the host-microbe metabolic interactions that maintain the balance of oxalate. Oxalate can enter the body either directly from the diet or indirectly as a terminal metabolite produced by the liver. Oxalate is produced by a number of commonly consumed plants such as rhubarb, tea, chocolate, sorrel, *Opuntia* cactus, saltbush, halogeton, the desert lily, and napier grass [32,34,47,48,49,50,51]. Insoluble oxalate produced as needle-like raphides by some plants may serve as a pre-ingestive defense to inhibit herbivory [48]. Soluble oxalate is more readily consumed and absorbed across the gut lining, and thus may serve as a post-ingestive toxin, potentially accumulating as calcium oxalate crystals in the kidneys. Both forms of oxalate are typically produced within plants [32]. Additionally, at least 13 dietary precursors, including glyoxalate, ascorbic acid, hydroxyproline, and glycine, are metabolized through complex pathways to produce NADH, while producing oxalate as a by-product [29,33]. Oxalate consumed directly in the diet can contribute greater than 50% of the oxalate excreted in the urine [15,30,35,52]. However, numerous factors contribute to urinary oxalate and typically, the endogenous production of oxalate may be the biggest contributor to urinary oxalate [35]. Elevated urinary oxalate is a risk factor for kidney stones [40,41,42]. The dietary contribution to the oxalate load in the body is illustrated by the fact that oxalate consumption averages 2.0 mmol/day in kidney-stone forming humans, while the total amount of oxalate excreted in the urine and feces averages 1.78 mmol/day [31]. The total excreted oxalate is a combination of both endogenous and dietary oxalate, after any microbial degradation or bioaccumulation.

The bioavailability of oxalate is mediated by the transport of oxalate across the GI tract [31,53,54,55]. The bulk of oxalate transport via absorption and secretion is mediated by trans-cellular anion transporters [56]. In particular, a number of transport proteins with affinity for oxalate have been identified from the SLC26 gene family and are primarily located in the small and large intestines [56]. Oxalate can also be passively absorbed in the stomach [56]. In rats where hyperoxaluria was experimentally-induced, the presence of particular bacteria in the colon can induce the secretion of oxalate into the GI tract [55]. The secretion of oxalate can lead to a net influx into the colon, where it can be further metabolized by the gut microbiota [55]. Oxalate that is not degraded by the microbiota can be excreted by the host in the feces or urine or can accumulate in the tubules and pelvis of the kidneys as calcium oxalate crystals. These crystals can aggregate to form kidney stones [31,42,57].

## 3. Microbial Oxalate Degradation

The tolerance of oxalate by mammals is often facilitated by the presence of oxalate-degrading bacteria in their gut microbiota [8,38,58,59]. One of the first oxalate-degrading gut microbes to be characterized was *Oxalobacter formigenes*. This species, which is common in the mammalian gut, requires oxalate as a carbon and energy source [60]. In addition, 18 other species have been identified to degrade oxalate but do not require it for growth. These include species from the genera *Lactobacillus*, *Bifidobacterium*, *Enterococcus*, and *Eubacterium* among others [61,62,63,64] (Table 1). One species, *Enterococcus faecalis*, may utilize oxalate as a sole carbon and energy source in an otherwise nutrient poor environment, but can utilize other substrates for growth as well [62].

**Table 1:** List of known oxalate-degrading bacteria commonly inhabiting the mammalian gut, where they were isolated, and their oxalate-degrading function. N/A means the pathway is unknown.

Organism	Source	Pathway	References
*Oxalobacter formigenes*	Various mammals	carbon/energy	[60,65]
*Eggerthella lenta*	Human stool	N/A	[61,66]
*Enterococcus gallinarum*	Woodrat feces	detoxification	[64]
*Enterococcus faecium*	Canine feces	N/A	[67]
*Enterococcus faecalis*	Human stool, canine feces	carbon/energy	[62,67]
*Provendencia rettgeri*	Human stool	N/A	[68]
*Streptococcus thermophilus*	Probiotic	detoxification	[63]
*Lactobacillus plantarum*	Probiotic, canine/feline feces	detoxification	[69,70]
*Lactobacillus gasseri*	Probiotic, woodrat gut	detoxification	[64,70]
*Lactobacillus casei*	Probiotic	detoxification	[70,71,72]
*Lactobacillus acidophilus*	Human stool	detoxification	[73]
*Lactobacillus rhamnosus*	Probiotic	detoxification	[70]
*Lactobacillus salviarius*	Probiotic	detoxification	[70]
*Lactobacillus johnsonii*	Woodrat gut	detoxification	[64]
*Bifidobacterium infantis*	Probiotic	detoxification	[63]
*Bifidobacterium animalis*	Human stool	detoxification	[70]
*Clostridium sporogenes*	Woodrat feces	detoxification	[64]
*Leuconostoc lactis*	Canine/feline feces	N/A	[69]
*Leuconostoc mesenteroides*	Canine feces	N/A	[72]

Microbial oxalate metabolism in the GI tract occurs via a well-described, two-step enzymatic reaction. In *O. formigenes*, the membrane-associated antiporter OxlT, mediates the simultaneous transfer of oxalate into the cell and formate out of the cell [73]. Once in the cell, oxalate is degraded by the microbial enzymes oxalyl-CoA decarboxylase and formyl-CoA transferase, which are produced by the genes *oxc* and *frc*, respectively [74,75,76,77]. Variants of the *oxc* and *frc* genes have been identified in some but not all oxalate-degrading gut bacteria, including those from the genera *Lactobacillus*, *Enterococcus*, and *Bifidobacterium* [62,70,78]. While these taxa do not have the OxlT gene, other gene products may mediate the transport of oxalate into the bacterial cell [78]. In the degradation of oxalate, one molecule of carbon dioxide and one molecule of formate are produced for every molecule of oxalate degraded [60].

While many of the oxalate-degrading bacteria in the GI tract share a common oxalate-degrading pathway, the purpose of oxalate degradation by the various taxa may be different. *Oxalobacter formigenes* requires oxalate as a carbon and energy source for growth and *E. faecalis* can also use it as a carbon and energy source [60,62]. However, species from the genera *Lactobacillus*, *Streptococcus*, and *Bifidobacterium*, are inhibited in growth with the presence of a high concentration of oxalate, but will degrade it when present [63,78]. Thus, some bacteria may co-opt the oxalate-degrading genes, which may have initially evolved as a means to extract carbon and energy from oxalate, to detoxify oxalate for themselves (discussed below).

## 4. The Gut Ecosystem Mediates Microbial Oxalate Degradation

The mammalian gut can be considered as a complex series of differentiated ecosystems with unique microenvironments among each gut region and also unique microniches within each region [6,21]. Four major sections can be described from various mammalian GI tracts: the foregut, stomach, small intestines, and hindgut. Foregut fermenters, such as cattle, goats, kangaroos, and sheep, have an enlarged foregut, which is the primary location of the gut microbiota [21,79,80,81]. Hindgut fermenting mammals such as horses, guinea pigs, wombats, and rabbits, have an enlarged hindgut, which is the primary site for the microbiota and house communities distinct from foregut fermenters [27,82,83]. The secretion and absorption of oxalate to and from the GI tract is differentiated among gut chambers, with the small intestines and proximal colon exhibiting net secretion and distal colon exhibiting net absorption [56]. Thus, the location of the oxalate degrading microbial community is key to understanding oxalate exposure in the host. Communities that occur prior to the small intestine such as those in the foregut, could significantly reduce oxalate exposure in the host; whereas communities concentrated in the hindgut may not. However, hindgut communities may reduce oxalate levels in the feces, which can be a source of food in copraphagic species [80]. 

The specific species and relative abundance of oxalate-degrading bacteria present within gut communities contributes to the community potential for oxalate degradation and the balance of oxalate in a mammalian host. Some species of oxalate-degrading bacteria, such as *O. formigenes* and *L. acidophilus*, can degrade large amounts of oxalate, while others, such as *L. casei*, degrade a fifth as much oxalate given the same conditions [70]. However, as the relative abundance of a particular species increases, so does its capacity to degrade oxalate within the whole community. For example, the white-throated woodrat (*Neotoma albigula*) is an herbivore that consumes a diet rich in oxalate. The *Lactobacillus* genus makes up 13% of the community in the woodrat foregut, while *Oxalobacter* makes up a much smaller proportion of 0.01%–0.02% [64]. Because *Lactobacillus* isolates from *N. abligula* degrade significant amounts of oxalate and have the *oxc* gene, this genus may be the dominant oxalate-degrading taxa in these mammals [64].

The environmental pH is an important factor that may facilitate the degradation of oxalate by the gut microbiota. *Lactobacillus acidophilus* increases the expression of the oxalate-degrading genes, *oxc* and *frc*, when the pH increases from 4.5 to 5.5, but expression is reduced at a pH of 6.8 [84]. Likewise, in *Bifidobacterium animalis*, a similar pattern of reduced *oxc* expression at moderate pH values is observed [78]. However, in *O. formigenes*, optimal oxalate degradation occurs at a pH of 6.4 [85]. This differentiation in optimal pH for oxalate degradation may permit unique oxalate-degrading niches to be distributed throughout the GI tract. For example, *N. albigula* harbors unique consortia of potential oxalate-degrading bacteria that are segregated between gut regions according to their optimal pH for oxalate degradation [64]. By filling multiple oxalate-degrading niches, the efficiency of oxalate degradation increases considerably and *N. albigula* can degrade greater than 90% of dietary oxalate consumed [47,86].

In addition to affecting oxalate degradation directly, the interaction between pH and oxalate exposure may have other wide-ranging effects on microbial community dynamics and function. In *L. acidophilus*, 315 genes are down-regulated with exposure to 1% oxalate at a pH of 6.8, while only 16 genes are up-regulated [84]. The oxalate-degrading genes *oxc* and *frc* are among those down-regulated under these conditions. Next-generation metagenomic techniques can assess how genes in whole microbial communities are affected by the flux of oxalate among gut regions with varying pH. This technique would allow for more accurate predictions of the function and community shifts of a given microbiota in response to oxalate. Work has begun in this area, particularly with the sequencing of the whole genome of *O. formigenes* as part of the human microbiome project (Broad Institute). Indicative of the importance of pH to oxalate degradation, the cyclic fatty acid configuration of *O. formigenes* suggests that this species has considerable acid tolerance [87].

Gut regions can be analogized with chemical reactors to further inform the microbial biotransformation of compounds [80]. For microbial oxalate degradation, oxalate is the reactant and the bacterial enzymes, oxalyl-CoA decarboxylase and formyl-CoA transferase, are the reagents. In batch-type reactors, similar to the rabbit cecum, the digesta is introduced in discrete batches with continuous stirring and the reactant decreases with increasing retention time [80]. In this case, the microbiota receives the greatest exposure to the reactant with the initial input, which decreases over time and leads to temporal heterogeneity in exposure. In continuous stir tank reactors (CSTR), such as the rumen of ruminants, digesta is completely mixed with a continuous flow of the reactant through the system, which maintains a constant concentration and rate of reaction [80]. In this type of gut, the complete mixing and uniform flow rate would ensure uniform exposure of the reactant to the reagents (microbial enzymes). Finally, in plug-flow reactors, like the colon of horses or humans, digesta flows continuously through a tube with little mixing [80]. Here, the concentration of the reactant is at its maximum upon entry and decreases along the length of the tube, which leads to spatial heterogeneity. However, the secretion of oxalate into the colon by *O. formigenes* can lead to a more uniform distribution of the reactant along the length of the colon [52].

The type of gut reactor facilitates the interaction between the reagents and the reactant in question. Oxalate would have the greatest impact on the microbiota in CSTRs, where the microbiota is exposed to a constant concentration with uniform mixing. In batch and plug-flow reactors, the impact on the microbiota would be lower because it is temporally or spatially segregated, respectively. If the microbiota biotransforms a toxic reactant to non-toxic by-products, as is the case with oxalate, then the greater the impact on the microbiota, the lower the impact of the toxin on the mammalian host. Thus, CSTRs, such as the rumen of ruminants, would be optimal for microbial toxin biotransformation.

## 5. Ecological Interactions within the Gut Microbiota in Response to Oxalate

The differential effects of oxalate exposure on gut microbial populations, in combination with the differentiated gut ecosystem, would produce complex microbe-microbe interactions. These interactions subsequently affect the concentration of oxalate in the gut and its bioavailability to the mammalian host. By classifying oxalate as a resource for some populations, like *O. formigenes*, and an inhibitor for others, such as *S. thermophilus*, a hypothetical model can be developed to understand the population dynamics of the gut microbiota as a whole *versus* a single taxon. This allows the generation of novel and testable hypotheses that can be explored with contemporary techniques.

**Figure 1:**
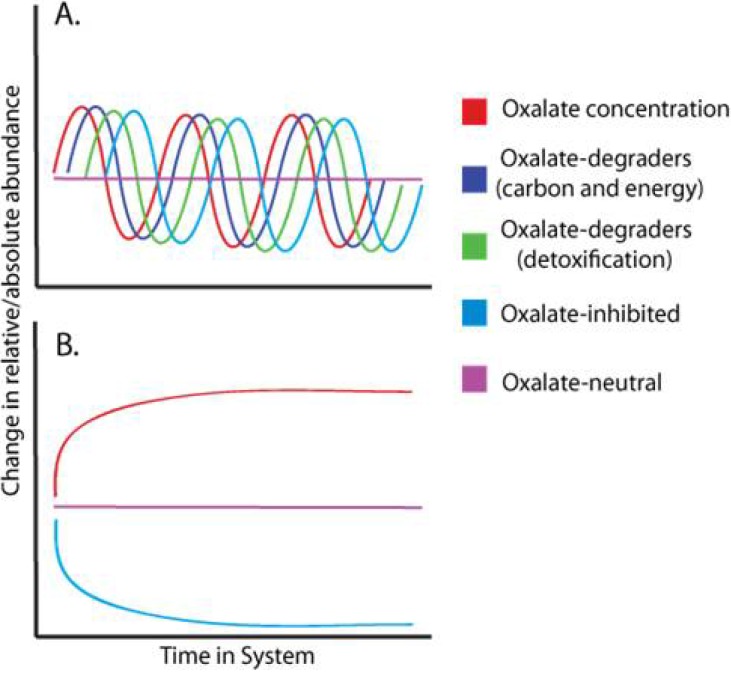
Hypothetical population/oxalate dynamics in the gut of a mammalian herbivore, with a regular influx of oxalate. (**A**) With a regular input of oxalate, the complex microbial community containing all four groups of bacteria will effectively degrade the presented oxalate. This would produce a relatively even community with high oxalate-degrading efficiency and minimal oxalate exposure to the host; (**B**) If oxalate-degrading bacteria are removed from the system, oxalate will become saturated and only be removed through host excretion. Those bacteria inhibited by oxalate would decrease to a minimal level. This system is seen in hyperoxaluric individuals and those vulnerable to repeated kidney stone formation. Oxalate-neutral bacteria would remain unchanged in either system.

The model identifies four groups of bacteria relative to oxalate usage. The hypothetical population dynamics of the four groups in response to a regular input of oxalate is summarized graphically in Figure 1. The first group of bacteria contains taxa that can use oxalate as a resource for growth. These bacteria would respond quickly to oxalate exposure to increase both relatively and absolutely within the microbiota. For example, in response to the addition of 0.6 mmol of oxalic acid directly to the rumen fluid of sheep, *O. formigenes* increased from 0.2% to 0.7% of the microbiota within seven days, corresponding to a four-fold increase in total DNA [88]. The second group of bacteria contains those taxa that are inhibited by oxalate, but can degrade it if present. This particular group may be more common than Group 1, and genera that fall into this group include *Bifidobacterium*, *Streptococcus*, and *Lactobacillus* [63,78]. As an example, *L. acidophilus* degraded 11.8% of oxalate in media containing 10 mM of oxalate over the course of three days, with a five-fold increase in population density, while at 20 mM of oxalate, this same species degraded 3.4%, with only a two-fold increase in population [63]. While at a disadvantage relative to bacteria that utilize oxalate for carbon and energy, Group 2 bacteria may benefit indirectly from oxalate exposure by being able to degrade it. Oxalate degradation would give them a competitive advantage over those bacteria that are inhibited by oxalate and cannot degrade it. In Group 3 bacteria, growth is inhibited in the presence of oxalate but they may indirectly benefit from the presence of other oxalate-degrading bacteria. While the presence of oxalate-degrading bacteria would allow Group 3 to grow in an otherwise inhospitable environment, they would be outcompeted by Groups 1 and 2 in the presence of high oxalate concentrations. Finally, Group 4 contains those bacteria that are unaffected by the presence of oxalate. Together, these four groups constitute all possible interactions that bacteria can exhibit in response to oxalate exposure with respect to growth rate.

While specific species have been identified as belonging to either Groups 1 or 2, more work is required to identify bacteria belonging to Groups 3 and 4 (Table 1). Changes in the abundances of individual taxa within whole communities can be tracked in response to oxalate using controlled, laboratory diet trials, coupled with next-generation sequencing techniques. Because individual groups within a complex microbiota are expected to exhibit unique responses (Figure 1), each taxon can be assigned to one of the four groups. Furthermore, whole communities lacking one or more of the groups would exhibit unique responses, such as those associated with hyperoxaluric individuals. This would potentially allow for the identification of individuals most likely to benefit from probiotics as well as the type of probiotic. For example, rats receiving inoculations of *O. formigenes* exhibited a constant and significantly lower level of urinary oxalate, consistent with Figure 1A [46]. In rats lacking oxalate-degrading bacteria and fed a 1.5% oxalate diet, the amount of oxalate excreted in the urine increased over a six-day period [46]. The increase of oxalate excreted over time is indicative of maladaptation of the microbiota in response to an increase in dietary oxalate load, and follows the model presented in Figure 1B. Reflective of differences in gut microbiota composition the addition of oxalate to the diet increases the oxalate degradation rates over time in some mammals, while in others the oxalate degradation rates remain low and unchanged [58,59,89,90]. Increases in oxalate degradation rates are likely the result of a rapid response by oxalate-degrading bacteria such as *O. formigenes* [88].

The microbial degradation of oxalate provides an opportunity to generate predictive mathematical models of microbial processes in complex communities. This effort would be relatively straightforward compared to degradation of other compounds given that it is a simple two-step enzymatic reaction within the gut microbiota with known reaction rates. Through the combination of molecular profiling and mathematical modeling, the population dynamics and microbe-microbe interactions associated with both short- and long-term exposure to oxalate could be predicted given a known microbial composition and input of oxalate. This information would not only inform the colonization and persistence of oxalate-degrading bacteria and activity within a host, but could also elucidate more complex biotransformation pathways. These are important in understanding how complex gut microbiota shapes host physiology [91]. 

## 6. Oxalate-Degrading Bacteria as Probiotics

There has been a concerted effort to introduce oxalate-degrading bacteria into the mammalian gut to alter ecosystem function towards more effective oxalate degradation and prevent disease [44,45,46,55,63,92]. The repeated use of certain antibiotics can result in the loss of naturally occurring oxalate-degrading bacteria [8,93,94,95,96,97]. With this loss, dietary and endogenous oxalate becomes more bioavailable both to the mammalian host and gut microbiota. Rats administered *O. formigenes* daily for two weeks exhibited a 39%–80% reduction in excreted urinary oxalate [46,55]. Likewise, humans administered *O. formigenes* for four weeks exhibited a 22%–92% reduction in excreted urinary oxalate [44]. However, in these studies, oxalate-degrading activity and the colonization of *O. formigenes* typically only persisted as long as there was either continuous inoculation, or maintenance of an oxalate-rich diet. In both rats and humans, activity and colonization is rapidly lost in as little as five days after returning to a low-oxalate diet [44,46,55]. Other studies used a mixed probiotic called “Oxadrop” (VSL Pharmaceuticals), which contains *L. acidophilus*, *L. brevis*, *S. thermophilus*, and *B. infantis.* Although Oxadrop taken with a normal diet did reduce oxalate excretion, when combined with a low oxalate diet, Oxadrop did not have an effect [63,92,98].

Researchers using oxalate-degrading bacteria as probiotics in the treatment of hyperoxaluria have demonstrated the potential for additional microbe-microbe metabolic interactions that indirectly support oxalate degradation. In contrast to individuals inoculated with probiotics, natural populations of oxalate-degrading bacteria persist in the gut, even after several generations on a low oxalate diet [49,52,55,58,97]. Oxalate-degrading bacteria in natural systems can increase in abundance to functionally relevant levels with increasing exposure to dietary oxalate [59,88,89,90,99]. This response illustrates the dynamic adaptability and shifting functional physiology associated with environmental fluctuations. The differences in persistence between natural oxalate-degrading populations and inoculated populations suggest that other microbe-microbe metabolic interactions facilitate the persistence of the oxalate-degrading function when dietary oxalate becomes scarce. 

An understanding of ecosystem dynamics is important in understanding the outcome when oxalate-degrading bacteria are given to humans as probiotics. With the administration of oxalate-degrading bacteria as probiotics, bacteria are introduced into a community that has adapted to the current level of oxalate intake. The presence of oxalate above these background levels, such as that on a high oxalate diet, gives the probiotic strains a competitive advantage. However, if this advantage is removed through a reduction in dietary oxalate, then the native community may quickly out-compete the probiotic strains. Thus, alternate approaches to introducing oxalate-degrading bacteria into the gut, which take into account complex metabolic and ecological interactions, may be required for successful inoculation.

Given the ecological interactions that occur within the gut microbiota in response to dietary oxalate, the use of whole communities adapted to oxalate degradation may be a more effective strategy for transferring oxalate-degrading function than isolated strains. Using whole communities ensures that all bacteria that maintain the oxalate-degrading function are present, increasing the probability of continued existence within the new community, even without an oxalate-rich diet or continued inoculation. Whole microbiota transplants are increasingly being used to treat a range of disorders including *Clostridium difficile* infection, irritable bowel syndrome, and inflammatory bowel syndrome [100,101]. In a systematic review of whole microbiota transplants used to treat *C. difficile* infections, the average success rate was 92% with a 4% death rate. The deaths were directly attributed to the infection itself [102]. While the mechanism responsible for the success of whole microbiota transplants has not yet been elucidated, it is thought to restore the normal function of the gut microbiota in individuals that receive the transplant [103]. Caution is advised before performing whole microbiota transplants across different species of animals. In transplants of the gut microbiota from zebrafish to mice and vice versa, the microbial lineages were the same between donor and recipient, but the relative abundances of the bacterial populations changed [104]. A similar phenomenon occurred in transplants of the gut microbiota from humans to pigs [105]. In should be noted that the recipients in these studies were germ-free prior to the transplant. Transplantation of microbes into animals that already have a native microbiota is considerably more complex. Thus, the efficacy of whole microbiota transfers must be assessed in terms of the resulting composition of the transplanted microbiota and the persistence of the transferred microbiota.

## 7. Evolutionary Considerations of Oxalate-Degrading Bacteria

The widespread presence of oxalate in the mammalian gut presents a large pool of bioavailable resources/toxins that can have a considerable impact on the gut microbiota, providing strong evolutionary pressure to develop mechanisms of oxalate utilization. Despite the toxicity of oxalate and the evolutionary capacity of mammals to metabolize a wide range of plant toxins, mammalian herbivores have not evolved a detoxification pathway to neutralize this toxin [43]. Instead, many mammals degrade the compound within their gut microbiota, circumventing the need to evolve their own enzymes for this function. The microbial sidestepping of the host’s own physiology has evolved at least one other time, through the microbial fermentation of cellulose [21,106]. 

The evolution of oxalate degradation among varied gut bacteria may have a common origin. The two groups of oxalate-degrading bacteria from the proposed model, Groups 1 and 2, largely use the same pathways, enzymes, and genes for degradation, but differ in oxalate usage. The gut ecosystem puts potentially thousands of interacting bacterial populations in close proximity of a resource- and toxin-rich environment. Thus, this ecosystem is one that not only exerts strong evolutionary pressures on the gut microbiota, but is highly conducive to horizontal gene transfer [107]. This includes genes that may have evolved to acquire resources, but which can be co-opted by other bacteria for detoxification. The most likely pathway for the proliferation of the *oxc/frc* genes would be the initial evolution of the genes in the Group 1 bacteria, such as *O. formigenes* that degrade oxalate for carbon and energy, with a later uptake by the Group 2 bacteria that degrade it for the purpose of detoxification. 

From an evolutionary perspective, the hypothesis that genes can be transferred from one microbe to another and co-opted for novel functions is transformative and worthy of further investigation. Horizontal gene transfer can have a considerable impact on the function of the whole community and ultimately the host phenotype [107]. For humans, a change in microbial community function could influence medical treatments and pharmaceutical drugs, many of which are biotransformed by the microbiota [91]. The advancement of personalized medicine could potentially be aided by understanding how genes are distributed among the microbiota and how they can be co-opted for novel functions [91]. 

## 8. Conclusions

The widespread production of oxalates in plants has considerable implications for the ecology and evolution of mammalian herbivores and their gut microbiota. The tolerance of oxalate can have a macroscale effect on mammalian hosts and a microscale effect on the microbial populations within the mammalian gut. This, in turn, contributes to the overall structure and function of the communities that develop. Mammalian tolerance of oxalate is driven by the microbe-microbe and microbe-host ecological interactions. Recent advancement of research into the gut microbiota of mammals has opened up the possibility of new and widespread evolutionary pathways that lead to rapid shifts in host physiology through acquiring novel microbial symbionts. In addition to the ecological and evolutionary implications of microbial-mediated host physiology, understanding these pathways may permit the ability of controlling physiological phenotypes associated with xenobiotic biotransformation, obesity, irritable bowel syndrome, and a number of others, through the use of targeted microbial additions or removals. Such an “ecological” approach to human health has the potential to be more effective and sustainable than traditional means and warrants continued exploration.
